# NEIGHBORHOOD AGE COMPOSITION AND SELF-RATED HEALTH: FINDINGS FROM A NATIONALLY REPRESENTATIVE STUDY

**DOI:** 10.1093/geroni/igz038.247

**Published:** 2019-11-08

**Authors:** Amanda Lehning, Amanda J Lehning, Nicole Mattocks, Kyeongmo Kim, Richard J Smith

**Affiliations:** 1 University of Maryland, Baltimore, Maryland, United States; 2 Virgina Commonwealth University, Richmond, Virginia, United States; 3 Wayne State University, Detroit, Michigan, United States

## Abstract

Neighborhood age composition is an understudied area. Furthermore, existing empirical and conceptual work is conflicting, with some indicating neighborhoods with more older adults are beneficial and other scholarship suggesting it can be detrimental. Using data from 7,197 older adults from the first wave (2011) of the National Health & Aging Trends Study combined with census tract data from the National Neighborhood Change Database, we examined the association between neighborhood age composition and self-rated health. Findings from logistic regression models indicate those living in neighborhoods with a growing concentration of older residents are significantly more likely to report lower self-rated health compared to those living in a neighborhood in which older adults overall are declining (β=1.51, p < .05) or are becoming diluted by younger residents (β=.66, p < .05). Results have implications for interventions promoting aging in place, particularly for those who may be stuck in place in age-concentrated neighborhoods.

